# Microbial Primer: Understanding the exponential growth of epidemics

**DOI:** 10.1099/mic.0.001723

**Published:** 2026-06-08

**Authors:** K.C. King, C.M. Saad-Roy, D. Coombs, B. Ashby

**Affiliations:** 1 Department of Zoology, University of British Columbia, Vancouver, Canada; 2 Department of Microbiology & Immunology, University of British Columbia, Vancouver, Canada; 3 Department of Biology, University of Oxford, Oxford, UK; 4 Department of Mathematics, University of British Columbia, Vancouver, Canada; 5 Department of Mathematics, Simon Fraser University, Burnaby, Canada

**Keywords:** epidemic, exponential growth, evolution, host population, pathogen

## Introduction

Exponential growth is a hallmark of many processes, including population growth, PCR amplification of DNA, nuclear fission and, crucially, epidemics. All infectious diseases have the potential to grow exponentially in susceptible host populations. Mathematical modelling has played an important role in characterizing pathogen growth dynamics across scales, both at within-host and population levels [1]. Yet, we generally tend to have poor intuition about exponential growth, consistently underestimating its compounding effects [2,3]. A lack of understanding about exponential growth can lead to the sudden and disastrous spread of infectious diseases both at a population scale and also within an infected host. Within a host, pathogens can replicate exponentially before immune responses curtail pathogen growth. In a population of hosts, new infections also grow exponentially during the early stages of an epidemic. The interactions of exponential growth across scales can lead to important implications for pathogen evolution and emergence (e.g. [4]).

Here, we focus on population-level exponential growth. We review fundamental aspects of how relatively simple mathematical quantities can tell us when an epidemic is likely to take off, when a new strain will replace an existing one, and when public health interventions will curtail an epidemic.

## The basic reproductive number in epidemiology and evolution

The *basic reproductive number* (R_0_) is one of the most important concepts in epidemiology. It represents the average number of new infections generated by a single infected individual in a population where everyone else is susceptible. R_0_ can be calculated directly in mathematical models or estimated from case data using a variety of methods (e.g. [5,8]). R₀ tells us whether an outbreak is likely to spread: if R₀ is greater than 1, each case produces more than one new case, and the epidemic will tend to grow. The size of R₀ also indicates how quickly an epidemic might grow when considered alongside the *serial interval* (the average time between symptom onset in an infected host and in the next host they infect).

Importantly, R₀ is not fixed. It reflects both the characteristics of the pathogen (e.g. influenza and measles have different R₀ values) and the population in which it spreads (e.g. measles can have an R₀ ranging from 12 to 18 depending on the population). All else being equal, the larger the R_0_, the faster an epidemic will spread. For instance, if R₀=2, then one infected host at the start of an epidemic will on average infect two others, who will each infect two more and so on (1 case becomes 2, then 4, then 8 etc.). This pattern is called *exponential growth*.

Individual hosts can vary in the degree to which they transmit pathogens. Key analyses following the 2002–2003 SARS outbreak revealed that there are often large heterogeneities in the risk of spreading but also in contracting an infectious disease [9]. Superspreading – where some individuals transmit infection far more than average – introduces substantial variation in transmission. This heterogeneity has two important consequences. First, it reduces the probability that an outbreak will occur because many infected hosts will have below-average transmission potential [9]. Second, if an epidemic does take off, superspreading can accelerate early epidemic growth. In such cases, clusters of high transmission can make case numbers rise more quickly than would be expected under more homogeneous transmission. Consequently, early estimates of R₀ based on initial case growth may be inflated if superspreading events are overrepresented in the data [9].

**Fig. 1.**
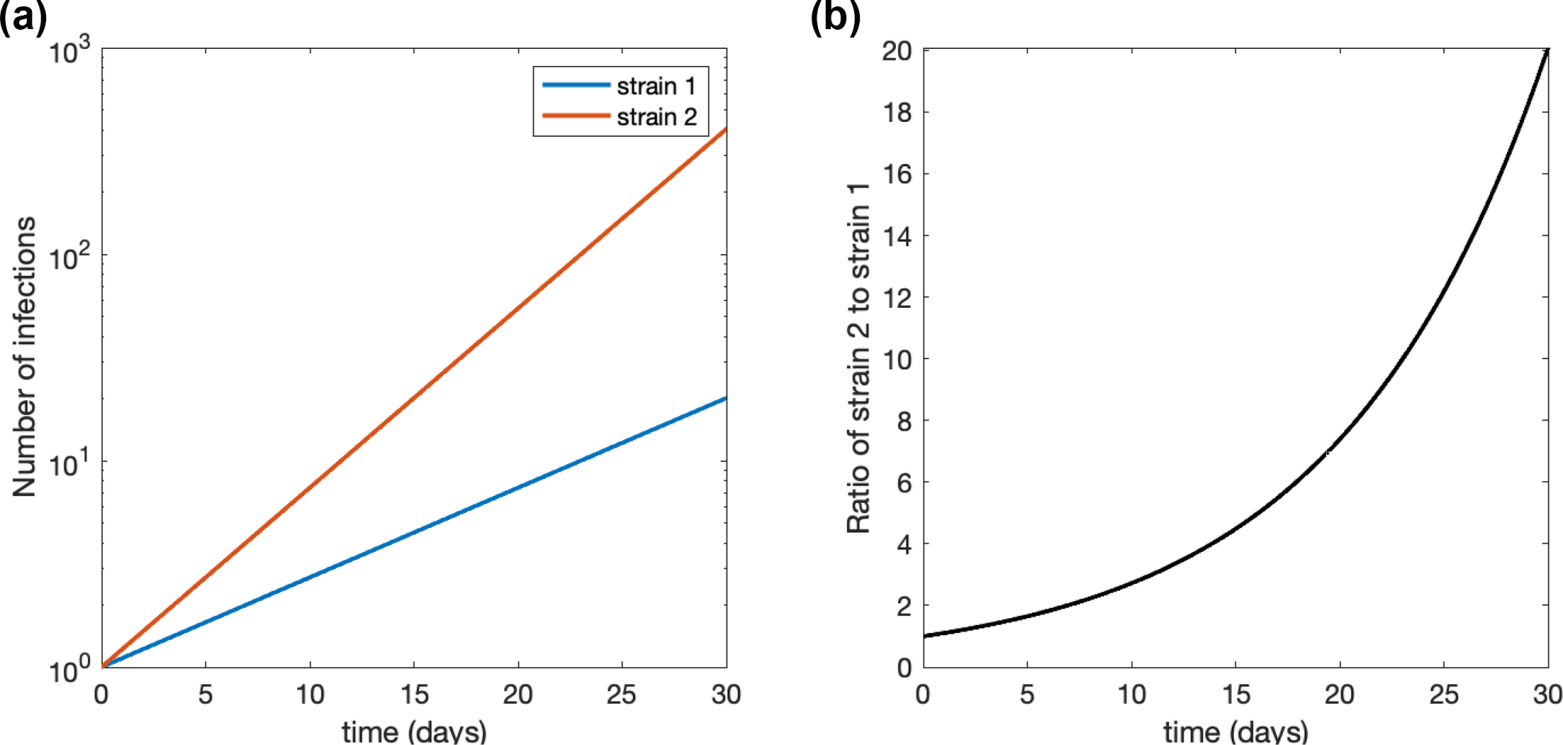
Both epidemiology and pathogen evolution are characterised by exponential growth. (**a**) Example of two pathogen strains with different R_0_ values (strain 1: R_0_=1.1, strain 2: R_0_=1.2) and the same average infectious period (1 day), leading to a *selection differential*. Note that exponential growth appears linear on a log scale. (**b**) A relatively small difference in growth rates is compounded due to exponential growth, leading to rapid replacement of one strain by another.

Exponential growth not only drives epidemics but also shapes pathogen evolution. Imagine two variants of a virus circulating in the same population. If one variant produces slightly more secondary infections than the other (for example, a R_e_ of 1.2 vs. 1.1), that small edge accumulates over time (Fig. 1). Just as with compound interest, the fitter variant grows more quickly and soon makes up a larger share of cases. This difference in fitness is known as a *selection differential –* the difference in growth rates between competing types.

In epidemiology, it explains why one pathogen variant can rapidly replace another. In evolutionary biology, the selection differential between pathogen strains determines which traits (e.g. high virulence or transmissibility) become more common across generations where they are endemic or emerging [10]. This pattern played out throughout the coronavirus disease 2019 (COVID-19) pandemic, as variants with greater transmissibility or partial escape from immunity quickly displaced their predecessors [11]. Selection differentials can also be affected by interventions that reduce overall transmission, such as social distancing or mask-wearing (see Day *et al*. [12] for epidemiological-evolutionary modelling and [13] for relevant data for SARS-CoV-2).

## Nothing lasts forever

Exponential growth cannot continue indefinitely, however. Imagine you choose two friends, each of whom chooses two more *unique* friends, and so on. At first, the numbers double each round (e.g. 2, 4, 8 and 16) but soon you run out of people who have not already been chosen. Some may then add only one new friend or none at all. Epidemics behave the same way: once enough people have been infected (or are no longer susceptible), the pool of susceptible individuals shrinks, and growth slows so that it is no longer exponential.

The effective reproduction number (R_e_) represents the average number of new infections caused by a single case at a particular point in time, rather than at the start of an epidemic (R_0_). If R_e_>1, the epidemic grows; if R_e_<1, it shrinks. Public health interventions such as vaccination, quarantine or social distancing therefore aim to reduce R_e_ below 1.

If R_e_ falls below 1 because enough people are immune, the population is said to have reached herd immunity [14]. During the course of an epidemic or through vaccination, immunity builds up in a host population, limiting the spread of an infectious disease. If the number of immune hosts is above the threshold for herd immunity, pathogen transmission slows to the point where the epidemic starts to shrink. The herd immunity threshold – the fraction of the population that must be immune for R_e_<1 – is ~1−1/R_0_ in simple models. Pathogens with higher R_0_, therefore, require higher levels of immunity (for example, smallpox at around 80%, measles at around 95%). Importantly, reaching herd immunity does not mean all hosts are safe from infection. Rather, it means that on average each case generates fewer than one new case, so the epidemic will tend to decline.

## Implications for controlling infectious disease spread and evolution

In exponential growth, the time it takes for cases to double stays the same, even as the epidemic becomes larger. Crucially, *exponential* does not necessarily mean *fast*. An epidemic can initially grow exponentially but still add cases at a modest pace. Imagine an empty Olympic swimming pool, which holds about 3.4 million liters of water. If you start with one drop of water (0.05 ml), then double the number of drops every 10 s (1, 2, 4, 8 etc.) and the pool will overflow in 6 min. Remarkably, it is still only about half full just 10 s before it overflows and is only about 1.5% full at 5 min.

In much the same way, an infectious disease may be rare during the early stages of an epidemic before it suddenly explodes. The real power – and problem – of exponential growth lies in this principle of a fixed doubling time. Indeed, biologists routinely use this fixed doubling time to their advantage when amplifying DNA through PCR. Much like PCR, where the doubling of a target section of DNA each cycle quickly leads to billions of copies, the exponential growth of an epidemic can rapidly grow out of control. Exponential growth also presents unique problems for fitting models to data, as small uncertainties in the data early on are amplified as the epidemic progresses, making accurate forecasts challenging.

Exponential growth means that early action is often necessary before an epidemic grows beyond the capacity of healthcare systems and before pathogens evolve to counter control measures. This assertion is also relevant to cases of emerging infectious diseases spreading in at-risk [15] and agricultural [16] populations, threatening biodiversity and food security, respectively. Early action can present socioeconomic or political challenges, as drastic measures may need to be taken at a population level even when the number of cases is low. Yet, delayed action can lead to significantly worse outcomes, as timely interventions can break chains of transmission and prevent exponential growth. One approach to limit exponential growth during early outbreaks may be to reduce the likelihood of superspreading by targeting interventions to individuals or groups who are at higher risk of transmission [17]. Effective communication about the nature of exponential growth in epidemics is essential for ensuring public support for significant interventions in public health, wildlife health and agricultural contexts.

## Abbreviations

R_0_: basic reproductive number
R_e_: effective reproduction number
